# Supplementary material for: Initial estimates of the minimal clinically important difference for the Neuropathic Pain Symptom Inventory: a systematic meta-analysis
Source: Front Pain Res (Lausanne). 2026 Jan 9;6:1637741. doi: 10.3389/fpain.2025.1637741 (PMC12827709; doi:10.3389/fpain.2025.1637741)
Supplement: Supplementary file 2 [file Table2.docx]

**Jeffrey Bower**
VP Analytics
Sana Health, Inc
2051 Dogwood St Suite 220
Louisville, CO 80027
Jeff@sana.io
951-236-3007

**05-28-2025**

*Frontiers in Pain Research*

**Subject:** Manuscript Submission: "Initial Estimates of the Minimal Clinically Important Difference (MCID) for the Neuropathic Pain Symptom Inventory (NPSI): A Systematic Meta-Analysis"

Dear Frontiers in Pain Research,

On behalf of myself and my co-authors, I am pleased to submit our manuscript, titled “Initial Estimates of the Minimal Clinically Important Difference (MCID) for the Neuropathic Pain Symptom Inventory (NPSI): A Systematic Meta-Analysis,” for consideration for publication in Frontiers in Pain Research. This work aims to provide the first distributional estimates of the MCID for the NPSI total score, addressing a critical gap in the interpretability of this widely used patient-reported outcome measure in neuropathic pain (NP) clinical trials and practice.

Neuropathic Pain (NP) is a multifaceted and chronic disorder that is diverse in causes and etiology that often causes considerable suffering and distress in patients. The Neuropathic Pain Symptom Inventory (NPSI) is commonly used in NP trials, yet a Minimal Clinically Important Difference (MCID) for its total score has not been established. An MCID would enhance the interpretability of NPSI scores, guiding clinicians, and researchers in assessing clinically important improvements in NP symptoms.

Neuropathic pain significantly impacts patients' quality of life, yet the absence of an MCID for the NPSI limits its utility in clinical trials and practice. By analyzing 17 treatment arms across 12 randomized controlled trials, we estimated MCIDs using three established distributional approaches and explored etiology-specific variations. Our findings offer clinicians and researchers an initial baseline for interpreting meaningful change in NPSI scores that was previously unknown. This work will support better-informed clinical trial designs and treatment evaluations.

We believe this manuscript aligns with *Frontiers in Pain Research’s* mission to advance the understanding and management of pain through rigorous, translational investigation. Our study offers actionable guidance for interpreting the NPSI and underscores the value of patient-centered metrics in evaluating treatment efficacy.

Key highlights of the manuscript:

- First distributional estimates of MCID for the NPSI total score.
- Rigorous meta-analytic approach spanning multiple NP etiologies.
- Practical recommendations for researchers and clinicians.

We affirm that the manuscript is original, has not been published elsewhere, and is not under consideration by another journal. Dr. Jeffrey Bower, one of the authors, is an employee of Sana Health Inc., but no conflicts of interest influenced the study design or interpretation of results.

Thank you for considering this submission. We look forward to the opportunity to contribute to *Frontiers in Pain Research* and would be happy to address any questions during the review process.

Sincerely,

**Dr. Jeffrey Bower**


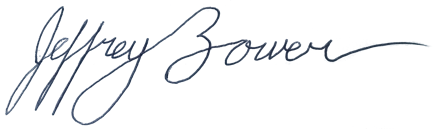


05-28-2025
